# Turner mosaic syndrome with Y chromosome material: characteristics, diagnosis, and management

**DOI:** 10.3389/fped.2026.1948778

**Published:** 2026-10-21

**Authors:** Tianyi Wang, Guangqi Zeng, Xu Cao, Hongliang Xia, Ting Zhang, Xiangming Yan, Mingcui Fu

**Affiliations:** 1 Department of Urology, Children’s Hospital of Soochow University, Suzhou, Jiangsu, China; 2 Department of Pediatric Surgery, Qinghai Provincial Maternal and Child Health Hospital, Xining, Qinghai, China

**Keywords:** DSD, gonadectomy, gonadoblastoma, pediatric, Turner syndrome

## Abstract

**Objective:**

This study investigated the clinical features and long-term outcomes of patients with Turner mosaic syndrome with Y chromosome material (TMSY), focusing on the prevalence of gonadoblastoma.

**Methods:**

Clinical data of children with TMSY were retrospectively collected, covering age, assigned sex, chief complaint, external genital phenotype, karyotype, FISH, sex hormones, imaging, age at gonadectomy, gonadal histology and prognosis.

**Results:**

Fifteen patients with TMSY were enrolled. The average follow-up was 47.33 months. All children were raised as females with female-like genitalia. Karyotyping diagnosed 9 (60%) patients, and FISH confirmed 6 (40%). 13 patients underwent gonadectomy, revealing gonadoblastoma in 4 (30.77%) cases, while 2 were monitored regularly. Growth hormone therapy was administered to 12 (80%) patients until they reached a satisfactory height.

**Conclusions:**

In our cohort, the prevalence of gonadoblastoma among patients with TMSY reached 30.77%. Given the favorable prognosis of gonadoblastoma, prophylactic gonadectomy is advised for patients with TMSY after a certain age to support spontaneous puberty and menstruation, enhancing patient involvement in clinical decisions.

## Introduction

In 1938, Dr. Henry Turner was the first to document seven cases of Turner syndrome (1). Turner syndrome represents the most prevalent sex chromosome anomaly in females, with an incidence rate of approximately 1 in 2,500 live births (2). Affected females typically exhibit clinical features such as short stature, lack of secondary sexual characteristics, primary amenorrhea, infertility, webbed neck, low posterior hairline, cubitus valgus, and either a missing or structurally abnormal second sex chromosome (3–5).

In Turner syndrome, 40%–50% of cases demonstrate a consistent 45,X monosomy in the blood karyotype, whereas 20% display a 45,X/46,XX mosaicism (6). Additionally, 30%–40% of individuals with Turner syndrome possess a structural abnormality in the second X chromosome, and approximately 10% exhibit a mosaic karyotype that includes Y chromosomal material (6–9). The detection of Turner mosaic syndrome with Y chromosome material (TMSY) can be further enhanced through the application of fluorescence *in situ* hybridization (FISH) technology (10). Children with TMSY are at an increased risk for developing gonadoblastoma, with reported incidence rates around 30% (11–14). Therefore, the detection of Y chromosome sequences in individuals with Turner syndrome holds substantial clinical importance. Nevertheless, the reported prevalence of gonadoblastoma can vary due to the limited number of patients with Turner syndrome mosaicism involving Y chromosome sequences (TMSY). This study was designed to elucidate the clinical characteristics and long-term outcomes of patients diagnosed with TMSY, as well as to evaluate the prevalence of gonadoblastoma within this cohort.

## Materials and methods

### Study design and participants

This research constitutes a retrospective, multi-center study incorporating follow-up investigations. Karyotyping was performed through classical cytogenetic analysis using peripheral blood lymphocytes or FISH technology. The inclusion criteria comprised a karyotype exhibiting mosaicism with 45,X/46,XY cells and a normal female phenotype without any ambiguity of external genitalia. We collected clinical data from 13 children diagnosed with TMSY who were admitted to the Department of Urology at the Children's Hospital of Soochow University between January 2016 and October 2023. In the interim, clinical data were collected from two patients diagnosed with TMSY who were treated at the Department of Pediatric Surgery, Qinghai Provincial Maternal and Child Health Hospital. No patients with TMSY at this institution underwent surgical intervention; instead, they were subject to outpatient follow-up with monitoring of gonadal development. This study received approval from the Medical Ethics Committee of Children's Hospital of Soochow University (2026CS165).

### Data collection and follow-up

The data included age, rearing sex, reason for initial consultation, external genital phenotype, karyotype and FISH results, sex hormone levels, imaging examinations, pathological findings, and prognosis. For patients with TMSY who are managed conservatively, annual monitoring involves the use of ultrasound to evaluate the uterus and ovaries, complemented by serum biochemistry, a sex hormone panel, and measurement of growth hormone levels.

### Statistical analysis

Data analysis was conducted using SPSS 26.0 statistical software (IBM Corporation, New York, United States). The normality of the data was assessed using the K-S test. For data not normally distributed, results are presented as median (interquartile range, IQR); otherwise, they are expressed as mean ± standard deviation. Categorical data are reported as percentages (%).

## Results

### Patient characteristics and diagnosis

A total of 15 patients with TMSY were included in the study. Of these, 13 underwent bilateral gonadectomy with salpingectomy, while 2 were managed with regular outpatient follow-up to monitor gonadal development. Demographic information, clinical outcomes, and gonadal histological characteristics were documented (Table 1). At the conclusion of the follow-up period, the mean age was 12.58 ± 3.02 years, and the mean duration of follow-up was 47.33 ± 30.78 months. At the initial presentation, the mean height was 124.39 ± 17.43 cm, increasing to 139.09 ± 17.76 cm by the final follow-up. All children were reared as females, exhibiting female-appearing external genitalia, and all were classified with a Prader grade of 0. No patients exhibited signs of hyperandrogenism, such as acne or hirsutism, nor any indications of virilization. The primary reasons for initial consultation were short stature or growth retardation, with no definitive family history reported in any of the patients. Three patients experienced menarche prior to undergoing gonadectomy. Ultrasonographic examination identified a rudimentary uterus and bilateral ovarian dysgenesis/streak gonads in all patients. Of the 15 patients with TMSY, 9 (60%) were diagnosed through peripheral blood karyotyping, while 6 (40%) were confirmed via FISH analysis (Table 1).

**Table 1:** Patients baseline features and clinical data in the 15 patients with turner mosaic syndrome with Y chromosome material (TMSY).

Case	Peripheral Karyotype	FISH	FSH (IU/L)	LH (IU/L)	E_2_ (pmol/L)	T (nmol/L)	AMH (ng/ml)	Puberty	Age at surgery(yr)	Pathology findings	Hormone therapy
1	45,X/46,XX	XY:70%,XO:15%,XXY:15%	11.90	0.25	52.58	<0.24	<0.07	N	10.08	bilateral gonadoblastoma	growth hormone
2	45,X/46,XX	XO:78%,XY:22%	41.88	1.95	43.31	<0.69	<0.07	N	9.83	bilateral streak gonads	growth hormone
3	45,X/46,XX	XO:60%,XY:33%,XYY:7%	16.46	17.04	32.82	<1.02	<0.07	N	11.50	bilateral streak gonads	growth hormone
4	45,X/46,XY	XO:62%,XY:38%	11.60	7.67	18.40	<0.60	<0.07	N	9.48	bilateral gonadoblastoma	growth hormone
5	45XO	XO:86%,XY:14%	34.70	8.90	29.40	<0.29	<0.07	N	10.50	bilateral streak gonads	growth hormone
6	45,X/46,XY	XO:47%,XY:54%	31.80	8.20	30.90	<0.16	<0.07	N	8.17	bilateral streak gonads	growth hormone
7	45,X/46,XY	XO:90%,XY:10%	87.50	14.20	32.40	<0.33	N/D	Y	10.50	bilateral streak gonads	follow-up
8	45,X/46,XY	N/D	45.20	5.30	12.59	<0.18	N/D	Y	12.58	Left gonadoblastoma right streak gonads	growth hormone
9	45,X/46,XY	N/D	N/D	N/D	N/D	N/D	N/D	N	9.17	bilateral streak gonads	follow-up
10	45,X/46,XX	N/D	N/D	N/D	N/D	N/D	N/D	N	10.25	bilateral streak gonads	follow-up
11	45,X/46,XX	XO:25%,XY:45%,XYY:30%	N/D	N/D	N/D	N/D	N/D	Y	11.96	bilateral streak gonads	growth hormone
12	45,X/46,XY	XO:70%,XY:30%	N/D	N/D	N/D	N/D	N/D	N	6.92	bilateral streak gonads	growth hormone
13	45,X/46,XY	N/D	N/D	N/D	N/D	N/D	N/D	N	4.17	bilateral gonadoblastoma	growth hormone
14	45,X/46,XY	N/D	9.00	48.60	44.00	<0.60	<0.07	N	N/D	N/D	growth hormone
15	45,X/46,XY	N/D	93.80	26.42	36.79	0.60	N/D	N	N/D	N/D	growth hormone

FSH, follicle stimulating hormone; LH, luteinizing hormone; E2, Estradiol; T, testosterone; AMH, anti-Müllerian Hormone; N/D, no data.

### Surgical management and histopathological findings

Gonadectomy was conducted on 13 patients (Figure 1), with the mean age at the time of surgery being 9.62 ± 2.22 years. Postoperative pathological analysis revealed gonadoblastoma in 4 patients (30.77%), of whom 3 exhibited bilateral gonadoblastoma and 1 presented with left-sided gonadoblastoma accompanied by right streak gonads. The remaining 9 patients (69.23%) exhibited bilateral streak gonads (Table 1).

**Figure 1:**
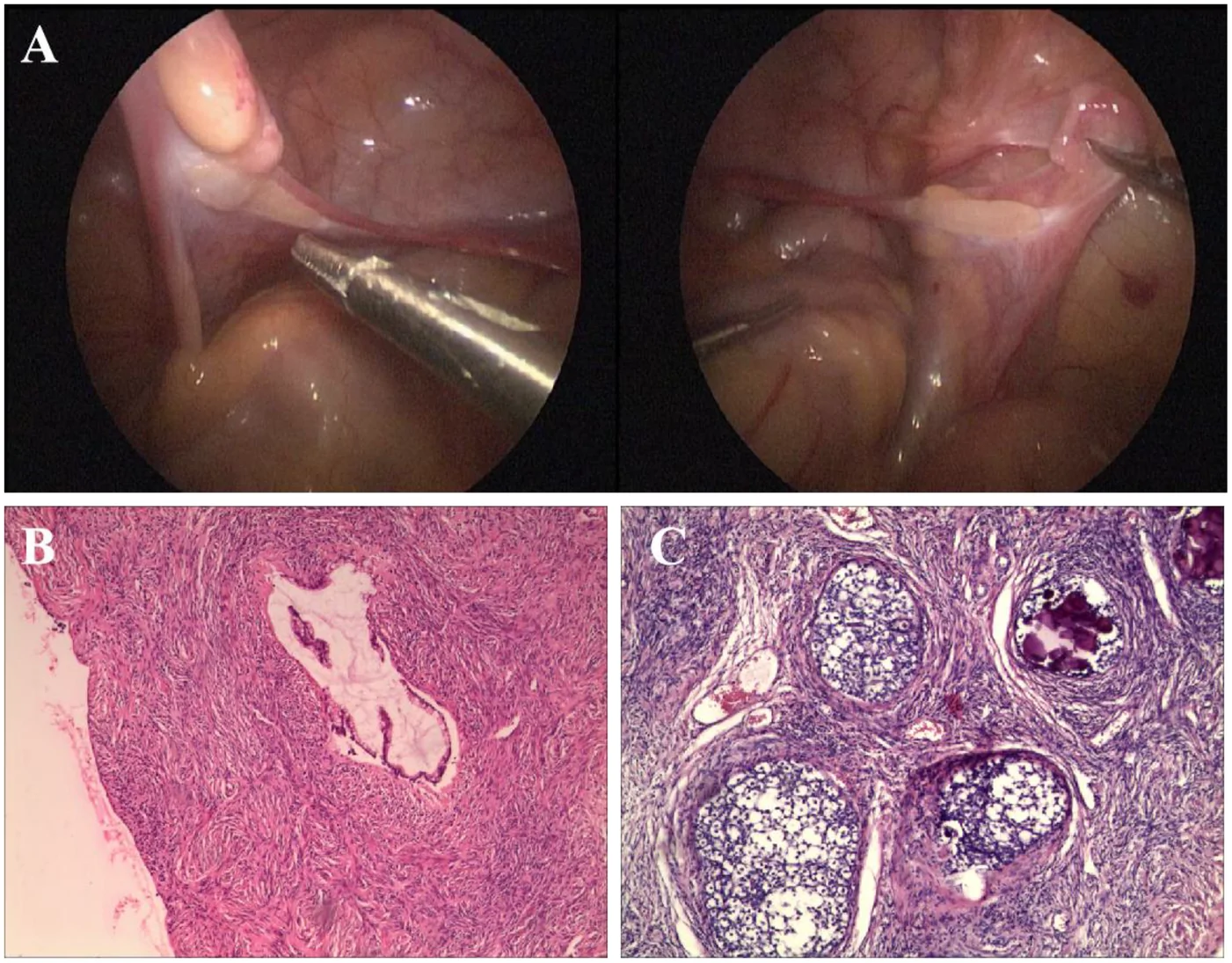
Intraoperative gross appearance and histopathological findings in patients with turner mosaic syndrome with Y chromosome material (TMSY). **(A)** Gross appearance of dysgenetic gonads during gonadectomy; **(B)** Pathological section of streak gonad; **(C)**: Pathological section of gonadoblastoma.

### Growth hormone therapy

Of the 15 patients, 12 (80%) underwent growth hormone therapy, initiated at a dose of 0.15 IU/kg/day, and continued until a satisfactory height was attained. Discontinuation of treatment may be considered for patients with limited growth potential, such as those with a bone age exceeding 14 years or a height increase of less than 2 cm per year following treatment.

## Discussion

Turner syndrome, although primarily characterized by the sex chromosome monosomy 45,X, can present with various mosaic karyotypes involving X and Y chromosomes in up to 50% of cases (15). The identification of Y chromosome material in Turner mosaic syndrome is clinically significant due to the associated risk of tumorigenesis, particularly gonadoblastoma (16). The risk of gonadal neoplasms is a critical factor in the management of patients with disorders of sex development (DSD). In this study, we conducted a retrospective review of 15 cases of TMSY and analyzed their clinical manifestations and long-term follow-up data from two participating medical centers.

Previous research has indicated that a low percentage of mosaicism may result from testing an insufficient number of cells or sampling an inadequate number of tissues. In our study, 9 patients (60%) were diagnosed with TMSY using peripheral blood samples, highlighting that conventional karyotyping may not be the most effective method for detecting cryptic Y chromosome material. Therefore, when structural abnormalities of sex chromosomes are identified, it is recommended to supplement with molecular cytogenetic tests, such as FISH techniques and/or array-based diagnostics (11). In this study, Y chromosome material was detected in 6 patients (40%) using the FISH assay.

Gonadoblastoma, a benign tumor first described by Scully in 1953, comprises undifferentiated cells similar to those found in gonadal development and includes sex-cord derivatives (17, 18). In 60% of cases, it may develop into an invasive dysgerminoma and can also progress to other malignant germ-cell tumor types (19). The 2006 consensus statement on Disorders of Sex Development (DSD) (20) recommends prophylactic gonadectomy for patients with bilateral streak gonads containing Y-chromosome material, as these gonads are non-functional in hormone secretion and lead to infertility. To date, there have been no documented cases of metastatic disease or mortality due to gonadal malignancy in individuals with TMSY (21, 22). There is significant variability in the reported incidence of gonadoblastoma among patients with TMSY. Morin et al. (23) reviewed 11 studies including 43 patients with TMSY and reported a gonadoblastoma incidence of 12%. Coyle et al. (24) conducted a retrospective analysis of data from three medical centers in Ireland, reporting a gonadoblastoma incidence of 28.6% (4 out of 14) among patients with TMSY. Another study from Argentina reported a gonadoblastoma incidence of 35.5% (7 out of 20) among patients with TMSY, with two cases pathologically confirmed as dysgerminoma (11). A Danish retrospective study reported a gonadoblastoma incidence of 10.0% (1/10) among patients with TMSY (13), while a Japanese retrospective study indicated a gonadoblastoma incidence of 57.1% (4/7) (15). In contrast, a single-center cohort from the Children's Hospital of Soochow University demonstrated an incidence rate of 30.77% (4/13).

Despite the general consensus advocating for prophylactic gonadectomy in patients with TMSY, the optimal timing for this surgical intervention remains a subject of debate. Clinicians must carefully balance the benefits and risks associated with the procedure. In assessing the risk and age of onset for gonadal tumors in patients with TMSY, it is crucial to consider the potential benefits of gonadal retention, such as the possibility of spontaneous puberty and fertility preservation. Gonadoblastoma typically manifests during the second decade of life; however, cases have been documented in children with gonadal dysgenesis as young as 5 months (24). In our study, the youngest patient diagnosed with gonadoblastoma was 6.17 yr, while the oldest was 12.83 yr. Based on our experience, preoperative endocrine evaluation of gonadal function is conducted, followed by a gonadal biopsy to confirm infertility. It is essential to measure a serum sex hormone panel, which include follicle-stimulating hormone (FSH), luteinizing hormone (LH), estradiol (E₂), testosterone (T), and anti-Müllerian hormone (AMH). When clinically warranted, additional testing should include the human chorionic gonadotropin (HCG) stimulation test and the luteinizing hormone-releasing hormone (LHRH) stimulation test. After obtaining guardian consent, dysgenetic streak gonads are removed. This study has limitations, including potential bias due to its retrospective nature and small sample size, and lack of molecular analysis on all tissues, leaving gonadoblastoma mechanisms unclear.

## Conclusions

In our cohort, the prevalence of gonadoblastoma in patients with TMSY was 30.77%. Prophylactic gonadectomy is recommended for patients with TMSY, but since gonadoblastoma has a favorable prognosis, gonadectomy can often be postponed with regular monitoring. This approach supports spontaneous puberty and menstruation, allowing patients greater involvement in clinical decisions.

## Data Availability

The original contributions presented in the study are included in the article/Supplementary Material, further inquiries can be directed to the corresponding author.
